# Effect of Selected Mercapto Flavor Compounds on Acrylamide Elimination in a Model System

**DOI:** 10.3390/molecules22060888

**Published:** 2017-05-31

**Authors:** Zhiyong Xiong, Bing Li, Lin Li, Liting Wan, Xiaolong Peng, Yongpo Yin

**Affiliations:** 1 School of Food Sciences and Engineering, South China University of Technology, Guangzhou 510640, China; xzygood2017@163.com (Z.X.); nbwlting@163.com (L.W.); kidsky4869@163.com (X.P.); yinyongpo@gmail.com (Y.Y.); 2 School of Chemical Engineering and Materials Science, Beijing Institute of Technology Zhuhai, Zhuhai 519088, China; 3 Guangdong Province Key Laboratory for Green Processing of Natural Products and Product Safety, Guangzhou 510640, China; 4 School of Chemical Engineering and Energy Technology, Dongguan University of Technology, Dongguan 523808, China

**Keywords:** acrylamide, 3-(alkylthio) propionamides, mercaptan, mercapto flavors, Michael addition

## Abstract

The effect of four mercapto flavor compounds (1,2-ethanedithiol, 1-butanethiol, 2-methyl-3-furanthiol, and 2-furanmethanethiol) on acrylamide elimination were investigated in model systems. The obtained results showed that mercaptans assayed were effective in elimination arylamide in a model system. Their reactivities for decreasing acrylamide content depended on mercaptan’s molecular structure and acrylamide disappearance decreased in the following order: 1,2-ethanedithiol > 2-methyl-3-furanthiol > 1-butanethiol > 2-furanmethanethiol. Mercaptans were added to acrylamide to produce the corresponding 3-(alkylthio) propionamides. This reaction was irreversible and only trace amounts of acrylamide were formed by thermal heating of 3-(alkylthio) propanamide. Although a large amount disappeared, only part of the acrylamide conversed into 3-(alkylthio) propionamides. All of these results constitute a fundamental proof of the complexity of the reactions involved in the removal of free acrylamide in foods. This implies mercapto flavor/aroma may directly or indirectly reduce the level of acrylamide in food processing. This study could be regarded as a pioneer contribution on acrylamide elimination in a model system by the addition of mercapto flavor compounds.

## 1. Introduction

Acrylamide (AA, CAS 79-06-1) as a α,β-unsaturated (conjugated) reactive molecule is widely used as a reactive monomer or intermediate in organic synthesis [1]. However, AA is a potential human carcinogen and genotoxicant and a known human neurotoxicant [2]. So it has been classified as a group 2A, “probably carcinogenic to humans”, chemical by the International Agency for Research on Cancer [3]. The European Food Safety Authority (EFSA)’s Expert Panel on Contaminants in the Food Chain (CONTAM) described dietary acrylamide as potentially increasing the risk of developing cancer for consumers in all age groups [4].

Acrylamide can be formed through the Maillard reaction during food thermal processing, especially starch-rich products such as fried potato, bread, potatoes and so on [5]. Extensive research show that reaction conditions such as reaction time and temperature play important roles in the formation of acrylamide. Maximum acrylamide were formed in glucose/asparagine model system when heated at 180 °C for around 4 min. [6]. There are 5–4000 ppb acrylamide in different food products, moderate levels in heated protein-rich foods and higher contents in carbohydrate-rich foods such as potato, beetroot, and selected commercial potato products [7]. Since acrylamide has been found to be produced in carbohydrate-rich foods as a result of the Maillard reaction [8], many food additives have been studied for their inhibitory role on acrylamide formation. Amino acids, proteins, ammonium hydrogen carbonates, sodium chloride, antioxidants, polyvalent cations, and other food/chemical additives have been reported to decrease the acrylamide content formed in thermally-processed snack and bakery foods and asparagine/sugar model systems [9,10,11,12,13]. However, once formed, acrylamide concentration is not always constant. As a α,β-unsaturated (conjugated) reactive compound, acrylamide can, thus, be trapped by reaction with numerous food constituents. The Michael reaction of some nucleophiles to the olefinic group of acrylamide could contribute to the loss of acrylamide. As commonly used nucleophilic groups in organic reactions, -SH, -NH_2_ present in the food ingredients may decrease acrylamide content in food [14].

Flavors provide not only cooked delicious but also tempting flavor/aroma notes to food products. In the food and beverage industry, flavors play a critical role in the appreciation of food and beverage products. Due to its extremely strong aroma, mercapto flavors are valuable flavor ingredient, and provide delightful food flavor/aroma, such as sesame oil, onion, garlic, and barbecue. Approved as a safe food additive by FEMA (Flavour Extract Manufacturers’ Association), mercapto flavors are often used to improve the flavor of food. Moreover, mercapto flavor compounds are also produced during food thermal processing by Maillard reaction as well as acrylamide. Thus, mercapto flavor/aroma may be added as food additive and also be produced in the thermal processing of food. Anyway, mercapto flavors and acrylamide may coexist in thermally-processed foods. However, as very good Michael donors, when they react with acrylamide, -SH is about 280 times more reactive than -NH_2_ at comparable reaction environments [15]. Therefore, mercapto flavors are likely to have an important effect on the concentrations of acrylamide in food.

This paper studies the effect of selected mercapto flavors to eliminate AA in model system in more detail: (i) 1-butanethiol, FEMA number 3478, maximum allowable usage: 0.02 ppm, a straight-chain mercaptan with beef, eggs, onion, garlic aroma [16]; (ii) 2-furanmethanethiol, number 2493, maximum allowable usage: 2.1 ppm, a great branched mercaptan with coffee, sesame oil, roasted aroma [17]; (iii) 2-methyl-3-furanthiol, FEMA number 3188, maximum allowable usage: 0.25 ppm, an aromatic mercaptan wih barbecue aroma [17]; (iv) 1,2-ethanedithiol, FEMA Number: 3484, maximum allowable usage: 0.2 ppm, containing two mercaptan groups, with onions, garlic, fragrant barbecue aroma [18]. In addition, this study analyzes the reaction between acrylamide and mercapto groups in an attempt to clarify some of the reaction pathways by which mercaptan, and their molecular structure, affect the acrylamide content determined in foods.

## 2. Results

### 2.1. Acrylamide Disappearance in Acrylamide/mercaptan Reaction Mixtures

Due to the inherent complexity of a food matrix, where many factors are involved that act one upon another, four mercaptans were employed to react with acrylamide under idealized conditions in a closed model system in order to evaluate the effect of mercapto flavors on the elimination of acrylamide. When heated in the presence of mercaptans, acrylamide was decreased to a certain degree. Corresponding adducts were observed and identified on the basis of their retention time and mass spectra determined by HPLC/MS using the synthesized adducts as a standard.

**Figure 1:**
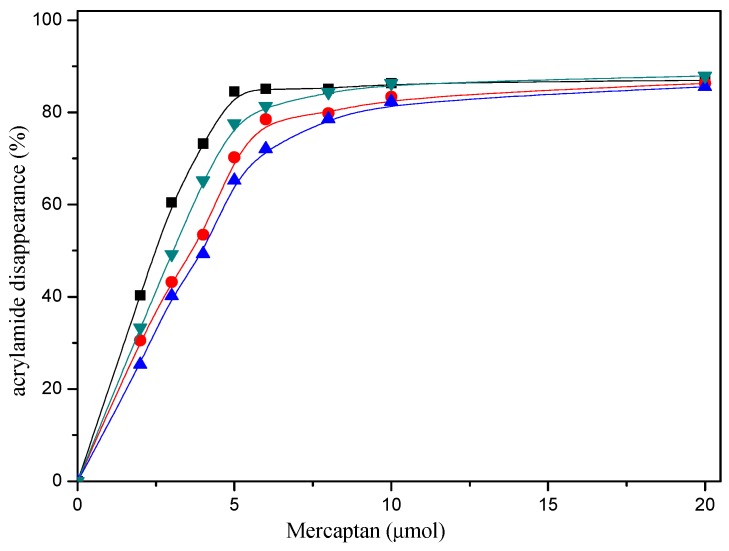
Dose-response effect of selected mercaptans on the acrylamide disappearance after heating an acrylamide (0.2 μmol)/mercaptans (0–20 μmol) model system at 180 °C and pH 7 for 30 min. Mercaptans assayed were 1,2-ethanedithiol (■), 1-butanethiol (●), 2-furanmethanethiol (▲), and 2-methyl-3-furanthiol (▼).

The reaction conditions and the amount of mercaptans added were responsible for the acrylamide disappearance. As can be seen from Figure 1, when the amount of mercaptans added was less than 5 μmol in the reaction mixture, acrylamide disappearance increased rapidly with mercaptans concentration increasing. However, acrylamide disappearance almost remained unchanged when the amount of mercaptans added was more than 10 μmol. Obviously, acrylamide disappearance increased as a function of the amount of mercaptans, and more than 80% acrylamide disappeared when 20 μmol mercaptans was added in the reaction mixture in model system. Nevertheless, the maximum allowable usage of mercapto flavors in food is more than the amount of acrylamide generally produced in food.

**Figure 2:**
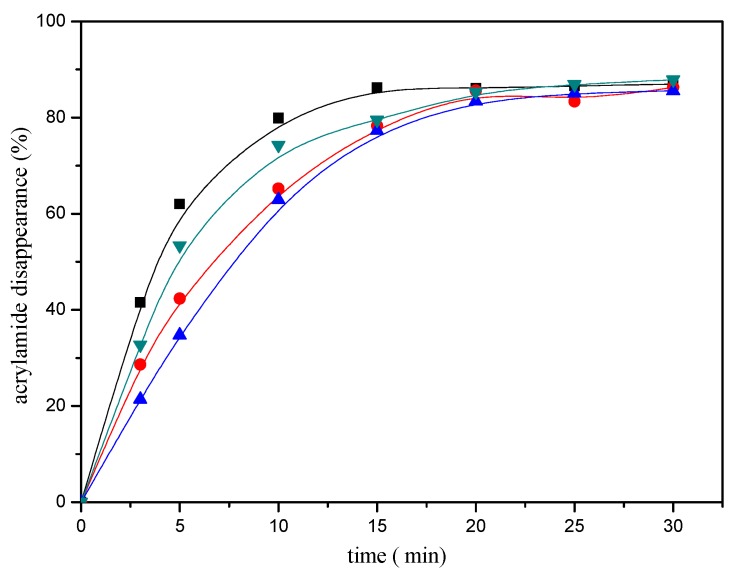
Time-response effect of selected mercaptans on the acrylamide disappearance after heating an acrylamide (0.2 μmol)/mercaptans (20 μmol) model system at 180 °C and pH 7. Mercaptans assayed were 1,2-ethanedithiol (■), 2-methyl-3-furanthiol (▼), 1-butanethiol (●), and 2-furanmethanethiol (▲).

**Figure 3:**
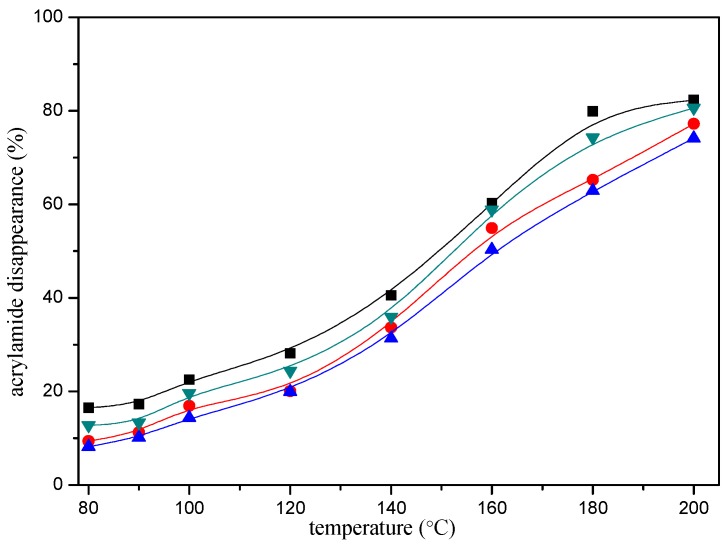
Temperature-response effect of selected mercaptans on the acrylamide disappearance after heating an acrylamide (0.2 μmol)/mercaptans (20 μmol) model system at pH 7 for 10 min. Mercaptans assayed were 1,2-ethanedithiol (■), 1-butanethiol (●), 2-furanmethanethiol (▲), and 2-methyl-3-furanthiol (▼).

In addition, the disappearance of the acrylamide depended on the reaction conditions. The heating time and temperature had a significant impact on the disappearance of the acrylamide. As shown in Figure 2, acrylamide disappearance increased rapidly and almost linearly as a function of time during the first ten minutes of the reaction. The disappearance of the acrylamide in the reaction mixture reached an apparent maximum after 20 min of heating at 180 °C, then tended to balance afterwards. The effect of the temperature as shown in Figure 3, acrylamide disappeared faster with the temperature increasing.

**Figure 4:**
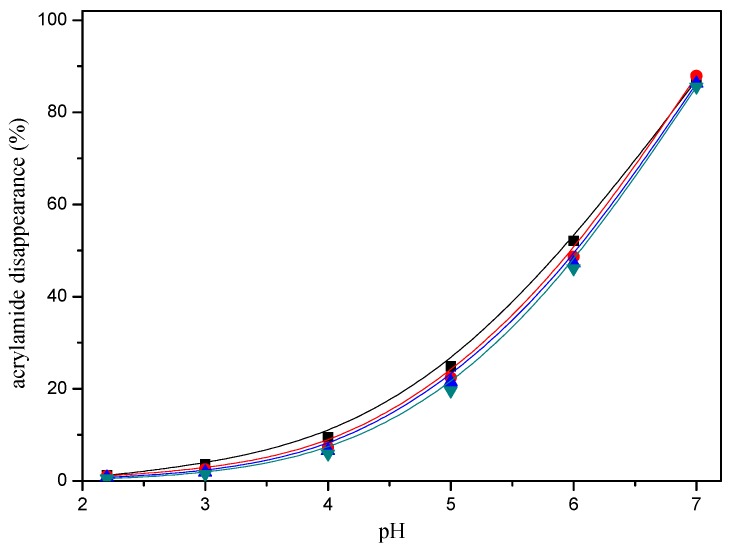
pH-response effect of selected mercaptans on the acrylamide disappearance after heating an acrylamide (0.2 μmol)/mercaptans (20 μmol) model system at 180 °C for 30 min. Mercaptans assayed were 1,2-ethanedithiol (■), 1-butanethiol (▲), 2-furanmethanethiol (▼), and 2-methyl-3-furanthiol (●).

Acrylamide disappearance also depended on the pH. Acrylamide disappearance increased slowly as the pH increased from pH 2.2 to 4, while this disappearance was faster when a higher pH was assayed from pH 5 to 7 in a model system (Figure 4).

### 2.2. Acrylamide Formation by Thermal Decomposition of Mercaptan-AA Adducts

**Figure 5:**
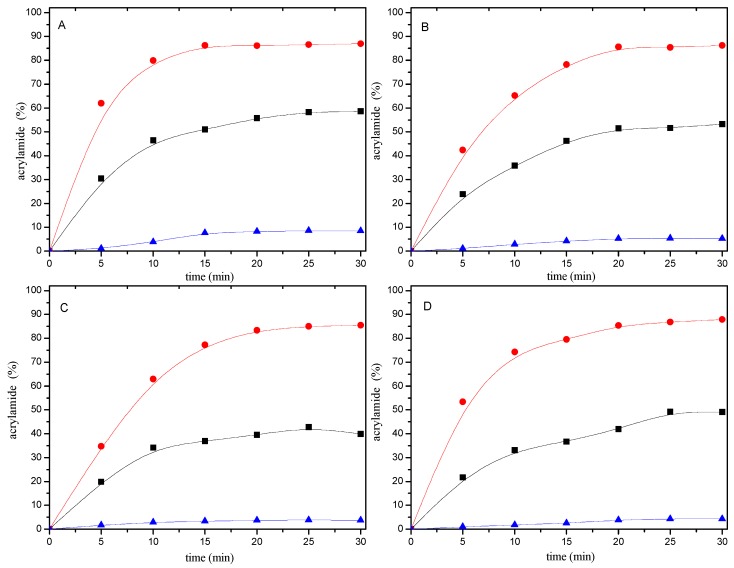
Time-response effect of selected mercaptans (**A**) 1,2-ethanedithiol; (**B**) 2-methyl-3-furanthiol; (**C**) 1-butanethiol; and (**D**) 2-furanmethanethiol on (●), the disappearance of acrylamide in acrylamide/mercaptan reaction mixtures; (■) the consumption of acrylamide produced to mercaptan-acrylamide adducts; (▲) the formation of acrylamide from mercaptan-acrylamide adducts. Reaction mixtures containing 0.2 μmol of either acrylamide or mercaptan-acrylamide adducts and 20 μmol of the mercaptan were heated at pH 7 and 180 °C for 0–30 min.

When 0.2 μmol of mercaptan-acrylamide adducts were heated at 180 °C for 0–30 min, they were stable and only trace amounts of acrylamide were formed (Figure 5). This shows that the addition reaction of the mercaptans to acrylamide were irreversible.

### 2.3. Acrylamide Consumption Produced to Mercaptan-Acrylamide Adducts

According to the amount of resulting adducts, it is easy to calculate the amount of acrylamide produced to mercaptan-acrylamide adducts. As can be seen from Figure 5, the consumption of acrylamide added to mercaptans also depended on the heating time. In the first ten minutes of the reaction, acrylamide disappearance and adducts formation increased linearly as a function of time. However, acrylamide disappearing faster than the speed of the conversion of acrylamide into adducts. In addition, the amount of acrylamide disappeared is much more than the amount of acrylamide which formed to adducts at the same reaction time.

### 2.4. Comparative Reactivity of Mercaptans for Acrylamide Removal

Figure 1 shows the effect of selected mercaptans on the acrylamide concentrations determined after heating acrylamide/mercaptan model system for 30 min at 180 °C. As observed in the Figure 1, acrylamide disappearance increased almost linearly as a function of the amount of the mercaptans added less than 5 μmol. By employing the disappearance rates obtained from the slopes of the lines, it was possible to concluded that the most reactive was 1,2-ethanedithiol, followed by 2-methyl-3-furanthiol and 1-butanethiol, 2-furanmethanethiol was the least reactive. Similar conclusions could be drawn from Figure 2.

Effect of Storage at Room Temperatures on the Acrylamide Content of Acrylamide/Mercaptan Mixtures.

The disappearance of acrylamide in acrylamide/mercaptan reaction mixtures was also observed at room temperature to study the effect of mercaptans on acrylamide elimination under storage conditions. After all, storage conditions of family cooking food have not been rigorously isolated from the air. Table 1 shows the acrylamide disappearance at room temperature. Acrylamide disappeared as a function of the storage time. It is noteworthy that 86.89% of the initial acrylamide had disappeared in acrylamide/1,2-ethanedithiol reaction mixture after six days. The concentration of acrylamide in the other three reaction mixture were also significantly reduced after 18 days.

## 3. Discussion

In recent years, acrylamide has received worldwide attention as a consequence of its potential toxicity at the concentrations formed during thermal food processing [19]. However, as an α,β-unsaturated amide, this toxicant is not a final product. It is a good Michael acceptor and may react with a variety of nucleophiles, such as -NH_2_ and -SH. The reactivity of acrylamide with various amino compounds in model reactions has been studied in recent years [20,21,22]. However, some studies had reported that cysteine was more effective to eliminate AA than other amino acids [23]. As very good Michael donors, -SH is likely to have an important effect on the concentrations of acrylamide in food. Mercaptans are often used in food as edible spices. Additionally, aroma components produced in the food thermal processing also contain a large number of mercapto compounds. These mercapto compounds may react with acrylamide during food processing. However, as a common food ingredient, this type of nucleophile is ignored in the literature and, generally, studies have focused on the effect of other compounds, such as amino acids and phenolic compounds, on the formation or the elimination of acrylamide [24]. Therefore, examining the effects of mercapto flavor has been quite poor. Under high temperatures (e.g., 180 °C), reactions in food are complex due to various food ingredients, in order to simplify the reaction, this study simulate the reaction system of food thermal processing to examine the fate of acrylamide when mercaptan spices is added. It should be noted that high concentrations of mercaptans have a very intense odor. Consequently, very small amounts of mercaptans are added in this experiment.

The present article confirms that mercapto compounds are efficient acrylamide scavengers in the model system. However, not all of the disappearing acrylamide converted to 3-(alkylthio)propionamide. Figure 5 shows the different reactions involved. The addition of mercaptans to acrylamide may proceed via a Michael addition pathway involving an ionic process or a free radical pathway. Generally, Michael addition reaction is based on activation of mercaptan by a base or activation of the acceptor olefins with Lewis acids. In order to avoid environmental impacts, several alternative solvents, such as ionic liquids [25], supercritical fluids [26], and water [27], as viable reaction media have been developed as efficient methodology for Michael addition reaction of mercaptans to unactivated alkenes. Possibly, water has a very specific role in Michael addition pathway through hydrogen bond formation with the sulfhydryl hydrogen of the mercaptans and, thus, increases the nucleophilicity of the mercaptan ion [27].

**Figure 6:**
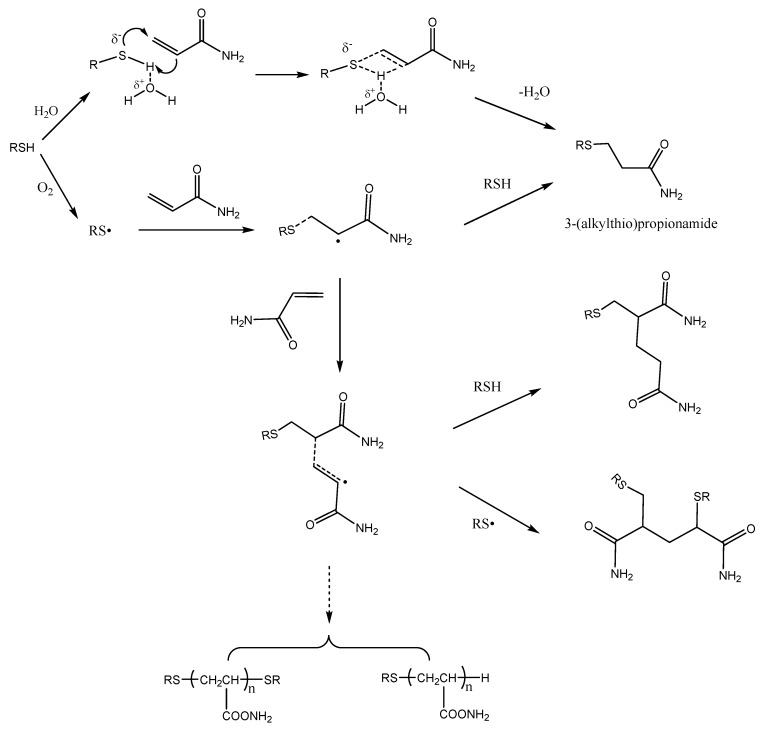
Proposed pathways for the disappearance of acrylamide in the presence of mercaptans.

The free-radical addition of mercaptans to acrylamide is a typical chain reaction. Mercaptan free-radicals are initiated by oxygen in the air, and subsequently add to the C=C of the acrylamide to form a carbon radical. The carbon radical then reacts with a mercaptan molecule to give the final product 3-(alkylthio)propionamide and a new thiyl radical, thus propagating the radical chain and then producing other polymers. It has been shown that the presence of oxygen in the atmosphere had a major role in the acrylamide disappearance in acrylamide/benzyl mercaptan reaction mixtures, while the presence of antioxidants prevented acrylamide losses [14]. This suggests that free radical addition is another way to eliminate acrylamide mercaptan. Figure 6 shows the different reactions involved.

However, the reactivity of the four mercaptans assayed for acrylamide removal is somewhat different. Acrylamide disappearance decreased in the following order: 1,2-ethanedithiol > 2-methyl-3-furanthiol > 1-butanethiol > 2-furanmethanethiol. Obviously, -SH plays a major role in this process. Thus, since there are two -SH groups in its molecule, 1,2-ethanedithiol is better than the other three mercaptans assayed in terms of reaction speed and elimination effect. In addition, the cleavage of an S-H bond is influenced by the structure of the mercaptans added. Thus, aromatic mercaptans are better active agents than aliphatic mercaptans, since in the former case the energy required to break the S-H bond is lowered by the conjugate stabilization of the thiyl radical or mercaptan ion formed. Therefore, 2-methyl-3-furanthiol is more reactive to react with acrylamide through Michael addition reaction or radical reaction. As for 1-butanethiol and 2-furanmethanethiol, they are all alkymercaptans with the least reactive in acrylamide elimination. However, the elimination activity of 2-furanmethanethiol is slightly weaker than 1-butanethiol, probably because there are many more side chains in the molecular of 2-furanmethanethiol. The steric effect makes it less active.

The impact of the compounds formed in these reactions on health remain to be investigated. The results obtained in this study suggest that some mercapto flavor have a good effect in eliminating acrylamide in food heat processing, and provide new evidence of the complexity of the reactions involved in the formation and elimination of acrylamide in foods.

## 4. Materials and Methods

### 4.1. Materials and Reagents

LC solvents were of HPLC grade (Honeywell, Seoul, South Korea). 1-butanethiol(≥98%), 2-furanmethanethiol (≥98%), Acrylamide (≥99.9%) was obtained from Sigma-Aldrich (Steinheim, Germany). 2-methyl-3-furanthiol(≥98%), 1,2-ethanedithiol(≥97%), was obtained from Aladdin (Shanghai, China). Labeled [1,2,3,-^13^C_3_]acrylamide was obtained from Cambridge Isotope Laboratories, Inc. (Andover, MA, USA). The solvents for NMR were CDCl_3_ and DMSO-*d*_6_ (≥99.9%) obtained from Sigma-Aldrich (Steinheim, Germany).

Mercaptan-acrylamide adducts were prepared by reaction of acrylamide with mercaptans. Briefly, acrylamide (1 mmol), mercaptans (2 mmol), Sodium hydroxide (0.4 mg) and methanol (10 mL) were added into a round-bottom flask. Then the mixture was stirred at room temperature in air, until acrylamide was completely consumed (checked by TLC). Afterwards, the pure product was obtained after purification by column chromatography or by recrystallization. The pure product was characterized by NMR spectroscopy on a 600 MHz spectrometer (Bruker, AVANCE 600, Karlsruhe, Germany).

*3-Butylsulfnylpropanamide* (**1**): ^1^H-NMR (600 MHz, CDCl_3_) δ = 0.92 (t, *J* = 7.4 Hz, 3H), 1.41 (dq, *J* = 14.6, 7.4 Hz, 2H), 1.63–1.52 (m, 2H), 2.51 (t, *J* = 7.2 Hz, 2H), 2.58–2.53 (m, 2H), 5.97 (s, NH), 7.29 (s, NH); ^13^C-NMR (600 MHz, CDCl_3_) δ = 13.64, 21.94, 27.50, 31.64, 32.06, 36.05, 173.97; HRMS: 184.0767 [M + Na]^+^.

*3-Furfurylthiopropanamide* (**2**): ^1^H-NMR (600 MHz, CDCl_3_) δ = 2.44 (t, *J* = 7.2 Hz, 2H), 2.80 (t, *J* = 7.2 Hz, 2H), 3.75 (s, 2H), 5.97 (s, NH), 6.21 (d, *J* = 3.2 Hz, 1H), 6.31 (dd, *J* = 3.2, 1.9 Hz, 1H), 7.29 (s, NH), 7.41–7.32 (m, 1H); ^13^C-NMR (600 MHz, CDCl_3_) δ = 27.29, 28.66, 35.71, 107.67, 110.56, 142.19, 151.43, 173.72; HRMS: 208.0401 [M + Na]^+^.

*3-[2-Methyl-3-furanthiol]-propanamide* (**3**): ^1^H-NMR (600 MHz, CDCl_3_) δ = 2.34 (s, 3H), 2.43 (t, *J* = 7.3 Hz, 2H), 2.89 (t, *J* = 7.3 Hz, 2H), 5.98 (s, NH), 6.34 (d, *J* = 1.9 Hz, 1H), 7.27 (s, NH) 7.28 (d, *J* = 2.0 Hz, 1H); ^13^C-NMR (600 MHz, CDCl_3_) δ = 11.81, 31.11, 35.74, 109.39, 114.87, 140.79, 155.18, 173.57; HRMS: 208.0407 [M + Na]^+^.

*3-[2-(2-Carbamoyl-ethylsulfanyl)-ethylsulfanyl]-propanamide* (**4**): ^1^H-NMR (600 MHz, DMSO-*d*_6_) δ = 2.33 (t, *J* = 7.3 Hz, 4H), 2.77–2.60 (m, 6H), 6.85 (s, NH), 7.35 (s, NH); ^13^C-NMR (600 MHz, DMSO-*d*_6_) δ = 27.36, 31.79, 36.15, 173.04. HRMS: 259.05456 [M + Na]^+^.

### 4.2. Acrylamide/Mercaptan Reactions in Aqueous Model System

Model reactions were carried out according to the methods of Zamora et al. [28] and Cai et al. [29] with slight modifications. Briefly, each 20-mL stainless-steel test tube contained 2 mL of 0.2 mol/L buffer solution (sodium citrate for pH 3–6 and sodium phosphate for pH 7–8) with 0.2 μmol acrylamide and different concentrations of mercaptans (0–20 μmol). The test tubes were capped with Teflon pad-filled stainless steel cap and the mixtures were heated at 80–180 °C in an oil bath installed with a magnetic stirrer for 0–30 min, or shaked at 180 rpm by means of a reciprocating water bath constant temperature oscillator for 0–18 day at room temperature. The assayed mercaptans were 1-butanethiol, 2-furanmethanethiol, 2-methyl-3-furanthiol, and 1,2-ethanedithiol.

After cooling (15 min at 0 °C), the test tubes were centrifuged at 2000 rpm for 10 min with a low-speed centrifuge (Jingli, Beijing, China). The reaction mixtures was decanted into 10-mL volumetric flasks. After mixing, 1 mL of the mixture were added into a 10-mL test tube, together with 10 μL of internal standard solution (1 mg/mL of labeled [1,2,3-^13^C_3_] acrylamide in methanol ), and then extracted with methanol (1 mL × 2). The extracts were studied by GC-MS for its acrylamide content. In addition, another 1 mL of the mixture was studied by LC-MS for the reaction products.

### 4.3. The Stability of Mercaptan-Acrylamide Adducts

Additionally, the formation of acrylamide in the thermal degradation of mercaptan-acrylamide adducts were also studied. This reaction was carried out analogously to acrylamide/mercaptan reactions. Thus, the mercaptan-acrylamide adducts (0.2 μmol) and corresponding mercaptan (20 μmol) were added into a stainless-steel test tube, together with 2 mL 0.2 mol/L sodium phosphate buffer, pH 7, and heated at 180 °C in closed test tubes for 0–30 min. After cooling, acrylamide was extracted as described above.

### 4.4. Preparation of Brominated Samples for the Analysis of Acrylamide

One milliliter of the extracts prepared by aforementioned pretreatment steps was treated with 100 μL sulfuric acid (10%, *v*/*v*), and then placed into refrigerating cabinet for precooling (0 °C, 30 min). Two-hundred microliters of 0.1 mol/L potassium bromate and 300 mg of potassium bromide powder, was added to the precooled solution. After shaking with a vortex blender, the reaction mixture was allowed to stand for 1 h at 4 °C. The excess of bromine was removed by addition of 1 mol/L sodium thiosulfate until the solution became colorless, and the solution was extracted with ethyl acetate (1 mL × 2), and the combined extracts were dried with sodium sulfate. One milliliter of the organic layer was evaporated until a volume of ~50 μL, treated with 10 μL of triethylamine, and analyzed by GC-MS.

### 4.5. Analysis of Acrylamide by GC-MS

The analysis of AA content was carried out analogously to the method of Zhang et al. [30] with slight modifications. GC-MS analyses were conducted with a Hewlett-Packard 6890 GC Plus coupled with an Agilent 5975 MSD (mass selective detector-quadrupole type) operated in selected ion monitoring (SIM) mode with positive electron impact (EI) ionization. HP5-MS capillary column (polysiloxane polymers, 30 m × Ø 0.25 mm, 0.25 m, J and W Scientific, Agilent, Santa Clara, CA, USA) was used for analytical separation. One microliter of the brominated sample was injected by the autosampler into the GC-MS system using the splitless flow control mode. Helium was chosen as the carrier gas at a flow rate of 1.0 mL/min. The temperature of the column oven was programmed as follows: isothermal at 60 °C for 1 min, increasing from 60 to 200 °C at a rate of 10 °C/min, and then subjected to isothermal conditions at 300 °C for 5 min. The temperatures of injector and transfer line to MSD were set at 250 °C and 280 °C, respectively. Analysis was performed using the electric impact mode at 70 eV. The SIM ions selected for identification of 2-bromopropionamide and 2-bromo (^13^C_3_)-propenamide were *m*/*z* 70, 149, and 152 and *m*/*z* 108, 150, and 152, respectively.

Following the whole procedure described above, quantification of acrylamide was carried out by preparing standard curves. Ten different concentration levels of acrylamide (0–150 μg) were used for each curve. Acrylamide content was directly proportional to the acrylamide/internal standard area ratio (*r* = 0.999, *p* < 0.0001). Data are mean values of, at least, two experiments. The coefficients of variation at the different concentrations were lower than 10%.

### 4.6. Analysis of Mercaptan-Acrylamide Adducts by HPLC/MS

HPLC/MS (Waters 1525, Waters Micromass ZQ, Milford, MA, USA) analyses of the reaction mixtures were carried out to identify the reaction products. The liquid phase conditions were as follows: separations were conducted on a Symmetry C18 (5 μm, 3.9 × 150 mm) analytical column. The mobile phase was methanol–water (30:70, *v*/*v*) at a flow rate of 0.5 mL/min. The mass spectrum conditions were as follows: the capillary voltage was 3.0 kV, the cone voltage was 20 V, the ion source temperature was 100 °C and the desolution temperature was 300 °C. SIR was conducted by operating the MS in ESI+ mode. Quantification of mercaptan-acrylamide adducts was carried out by preparing standard curves of the purified product prepared above.

### 4.7. Statistical Analysis

Statistical analysis was performed by using Student’s *t*-test with SPSS 19.0 software (IBM Corporation, Armonk, NY, USA). Analysis of variance (ANOVA) was tested on a significance level of *p* < 0.05.

## Figures and Tables

**Table 1:** Effect of storage at room temperature on acrylamide disappearance ^a^.

Time (Days)	1,2-Ethanedithiol	2-Methyl-3-furanthiol	1-Butanethiol	2-Methyl-1-butanethiol
0	−0.04 ± 3.10 ^b^	−0.60 ± 2.68 ^b^	0.16 ± 3.58 ^b^	0.19 ± 3.66 ^b^
3	78.01 ± 3.04 ^c^	55.94 ± 2.12 ^c^	30.74 ± 1.26 ^c^	29.44 ± 2.96 ^c^
6	86.89 ± 2.12 ^d^	73.85 ± 3.73 ^d^	57.02 ± 2.30 ^d^	56.46 ± 1.24 ^d^
9	90.45 ± 1.06d ^e^	87.52 ± 0.86 ^e^	70.60 ± 0.72 ^e^	68.19 ± 0.30 ^e^
12	92.08 ± 0.21 ^ef^	90.61 ± 1.47 ^ef^	81.05 ± 1.92 ^f^	80.13 ± 2.05 ^f^
15	93.21 ± 2.52 ^ef^	91.29 ± 1.59 ^f^	82.43 ± 1.75 ^f^	81.19 ± 3.67 ^f^
18	94.61 ± 0.19 ^f^	92.22 ± 0.19 ^f^	84.29 ± 3.11 ^f^	80.83 ± 0.41 ^f^

^a^ Values are acrylamide disappearance (%) in a model system containing 0.2 μmol of acrylamide and 20 μmol of mercaptans. Values are mean ± SD for, at least, two independent experiments. Means with different letters in the same column are significantly different (*p* < 0.05).
